# Bovine Papillomavirus in Brazil: Detection of Coinfection of Unusual Types by a PCR-RFLP Method

**DOI:** 10.1155/2013/270898

**Published:** 2013-06-24

**Authors:** R. F. Carvalho, S. T. Sakata, D. N. S. Giovanni, E. Mori, P. E. Brandão, L. J. Richtzenhain, C. R. Pozzi, J. R. P. Arcaro, M. S. Miranda, J. Mazzuchelli-de-Souza, T. C. Melo, G. Comenale, S. L. M. R. Assaf, W. Beçak, R. C. Stocco

**Affiliations:** ^1^Laboratório de Genética, Instituto Butantan, Secretária de Estado da Saúde, Avenida Vital Brasil 1500, Butantan, 05503-900 São Paulo, SP, Brazil; ^2^Laboratório de Biologia Molecular Aplicada e Sorologia, Departamento de Medicina Veterinária Preventiva e Saúde Animal, Faculdade de Medicina Veterinária, e Zootecnia da Universidade de São Paulo, Avenida Prof. Dr. Orlando Marques de Paiva, 87 Cidade Universitária, Butantan, 05508-270 São Paulo, SP, Brazil; ^3^Centro de Pesquisa em Pecuária do Leite, Instituto de Zootecnia, Agencia Paulista de Tecnologia dos Agronegócios, CAPTA Bovinos Leiteiros, Rodovia Luiz de Queiroz km 129, 13460-000 Nova Odessa, SP, Brazil; ^4^Programa de Pós-Graduação Interunidades em Biotecnologia, Instituto de Ciências Biomédicas, Edifício ICB-III, Universidade de São Paulo, Avenida Prof. Lineu Prestes, 2415 Cidade Universitária, Butantan, 05508-900 São Paulo, SP, Brazil; ^5^Programa de Pós-Graduação em Biologia Estrutural e Funcional, Universidade Federal de São Paulo, Rua Botucatu, 740 Vila Clementino, 04023-900 São Paulo, SP, Brazil; ^6^Departamento de Biologia, Universidade Federal da Integração Latino-Americana (UNILA), Avenida Tancredo Neves 6731, Bloco 4, 85867-970 Foz do Iguaçú, PR, Brazil

## Abstract

Bovine papillomavirus (BPV) is recognized as a causal agent of benign and malignant tumors in cattle. Thirteen types of BPV are currently characterized and classified into three distinct genera, associated with different pathological outcomes. The described BPV types as well as other putative ones have been demonstrated by molecular biology methods, mainly by the employment of degenerated PCR primers. Specifically, divergences in the nucleotide sequence of the L1 gene are useful for the identification and classification of new papillomavirus types. On the present work, a method based on the PCR-RFLP technique and DNA sequencing was evaluated as a screening tool, allowing for the detection of two relatively rare types of BPV in lesions samples from a six-year-old Holstein dairy cow, chronically affected with cutaneous papillomatosis. These findings point to the dissemination of BPVs with unclear pathogenic potential, since two relatively rare, new described BPV types, which were first characterized in Japan, were also detected in Brazil.

## 1. Introduction

The Bovine papillomavirus (BPV) is recognized as the causal agent of benign and malignant tumors in cattle, such as cutaneous papillomas, benign fibroplasias, urinary bladder, and esophagus cancer, causing significant economic losses. This oncogenic virus has a double-stranded circular DNA genome of approximately eight kilobases [1].

Currently, the *Papillomaviridae* family is divided into 16 genera according to their genomic organization [2, 3]. The papillomavirus (PV) genome codified functional, early (E) proteins and structural, late (L) proteins, expressed at different stages of the viral cycle. The L1 is the most conserved gene within a PV genome and has therefore been used for the identification of new PVs: one PV isolate is recognized as a new type if the complete genome has been cloned and the DNA sequence of L1 differs by more than 10% from the closest known PV type. Differences between 2% and 10% define a subtype and less than 2%, a variant [3]. Thirteen types of BPVs are currently well characterized and classified into three distinct genera—Delta, Epsilon, and Xi—each one associated with epithelia lesions of specific histological nature [4].

The BPVs-1 and -2 are classified as *Delta papillomaviruses *[5]. Characteristically, these types induce the appearance of fibropapillomas, associated with the recruitment of the subepithelial fibroblasts [6]. As far as concerned, both types are also unique in their ability to infect different host species, not only bovines, causing the equine sarcoid [7]. Lately, the genome of a new Delta-BPV type (BPV-13) was fully sequenced [8].

A larger number of BPV types (-3, -4, -6, -9, -10, -11 and -12) belong to the *Xipapillomavirus* genus. These viruses are considered exclusively epitheliotropic, inducing the formation of “true papillomas,” without the involvement of fibroblasts [9–11]. On the other hand, the BPVs-5 and -8 have the potential to induce both fibropapillomas and true papillomas in the course of their infectious cycle, being classified into a third genus, *Epsilonpapillomavirus* [12, 13]. The BPV-7 represents an exception and is classified separately (unsigned genus). This virus was first isolated from a cutaneous papilloma lesion and also from healthy teat skin samples [14].

The thirteen described BPV types as well as other putative ones have been demonstrated by molecular biology methods, since papillomaviruses are not prone to be replicated or recovered in cell cultures [15–19]. According to the guidelines outlined by the Papil omavirus Nomenclature Committee (14th International Papillomavirus Conference, Quebec City, QC, Canada), it has been specified that the amplified sequences isolated from novel papillomaviruses isolates could indicate only putative new PV types—instead of PV types—since the PCR amplicons represent only part of the L1 gene [15].

The use of PCR assays with degenerated primers, followed by sequencing has allowed the identification of several PV types in human and other animal hosts [15, 20]. The PCR primer FAP set was designed from two relatively conserved regions found in the L1 gene and has been shown to amplify PVs DNA from both papillomas and healthy tissue of many animal species, including BPVs in bovines [15, 16, 20].

Brazil has a cattle herd of approximately 210 million, being a major exporter of meat, milk and leather. BPVs have been previously detected in Brazil [21], but the extent of the impact of BPVs associated diseases, both in dairy and cattle herds, needs further studies. Available reports in different regions of the country indicate a significant diversity of viral types among the Brazilian herd, implying an evident disease burden [18, 22–25]. Thus, the improvement of knowledge concerning the diagnosis and related clinical aspects of different BPV types among the Brazilian herd should be considered in the development of new sanitary measures, aiming to the prevention of BPV infection and its consequences. 

Unfortunately, BPV epidemiological surveys are still limited by the availability of high-throughput diagnostic techniques that could discriminate different BPV sequences at the same time in co infected samples [16, 26]. In this context, the present work represents an effort to identify BPV types employing an alternative screening method based on the PCR-RFLP technique and correlating the histological data of the analyzed lesions with the diagnosed viral type.

## 2. Material and Methods

*In silico generation of RFLPs*: the L1 FAP segment digestion profiles of the BPVs-1 to -13 could be generated with NEB cutter 2.0 [27] from all L1 complete nucleotide sequences available in Genbank (http://www.ncbi.nlm.nih.gov/genbank/). The restriction enzymes sites were chosen both by its presence (or absence) as well as the generated digestion fragments sizes, in order to differentiate those thirteen different BPV types. *Histopathological analysis*: wart biopsies were obtained from the trunk of a six-year-old Holstein dairy cow chronically affected with cutaneous papillomatosis. Samples from three different lesions were submitted to macroscopic, histological (hematoxylin and Eosin staining) and molecular analyses. *DNA extraction and PCR*: DNA was extracted from warts for viral typing (Illustra tissue and cells genomic Prep Mini Spin GE Healthcare) and an approximately 470, base-pairs L1 gene segment was amplified using the following primer sequences: forward: FAP59 (5′-TAA CWG TIG GIC AYC CWT ATT-3′) reverse: FAP64 (5′-CCWATATCWVHCATITCICCATC-3′). The PCR, were performed with slight modifications of a previously described protocol [16]. In detail the amplification reactions were performed in a Corbett CG1-96 thermocycler (Corbett Life Science, Sydney, Australia), with GoTaq Master Mix (Promega, Madison, USA), under the following conditions: 5 min at 95°C, followed by 35 cycles of 1 min and 30s at 95°C, 2 min at 52°C and 1 min and 30s at 72°C and a final extension step of 5 min at 72°C. *Restriction analysis*: an aliquot of PCR fragments were submitted to digestion reactions for RFLP analysis with four different restriction enzymes (*Dde*I*, Hinf*I*, Hind*III*, MsL*I), following manufacturer's instructions (New England Biolabs, Ipswich, USA). Cloned BPV-1 and BPV-2 genomes as well as a known typed clinical sample (Mg-19, BPV-2 typed) were used as positive controls. PCR-RFLP products were analyzed in 2.0% agarose gel electrophoresis stained with ethidium bromide (0.5 *μ*g/mL) in TAE buffer and visualized under UV light. *Sequencing*: An aliquot of all generated PCR fragments were purified with extraction columns (Illustra GFX PCR DNA and Gel Band Purification Kit GE Healthcare). DNA concentration and purity were determined in a spectrophotometer (Eppendorf BioPhotometer, Hamburg, Germany) and submitted to sequencing reactions: three independent sequencing reactions were done for each PCR fragment in an ABI377 PRISM Genetic Analyzer (Life Applied Biosystems, USA). The quality of DNA sequences was checked and overlapping fragments were assembled using the BioEdit package 7.0.9.0 [28]. Assembled sequences with high quality were aligned using ClustalW 1.83 [29] with default gap penalties. Homology analyses were performed with the NCBI database and BLAST [30]. BioEdit software was used to identify the equivalent amino acid sequences. The sequence alignments were performed using the MEGA 5.0 software [31], using full alignment and 2000 total replications on the bootstrap, in order to ensure a higher level of confidence to our analysis [32]. *Phylogenetic Analysis*: phylogenetic relationship comparing nucleotide sequences was performed with MEGA. Neighbor-joining trees were drawn using TreeView version 1.6.6 [33]. Nucleotide and amino acid sequences from other BPV types and of a HPV-16 were retrieved from the GenBank (http://www.ncbi.nlm.nih.gov/) for comparison with the obtained sequences here. 

*Ethics Statement.* The protocols used in this study were approved by the Ethical Committee for Animal Experimentation of the Instituto de Zootecnia (Protocol no. 109, on July 06th, 2009) assigned by the President of this Committee. All efforts were made to minimize animal suffering.

## 3. Results

**Figure 1:**
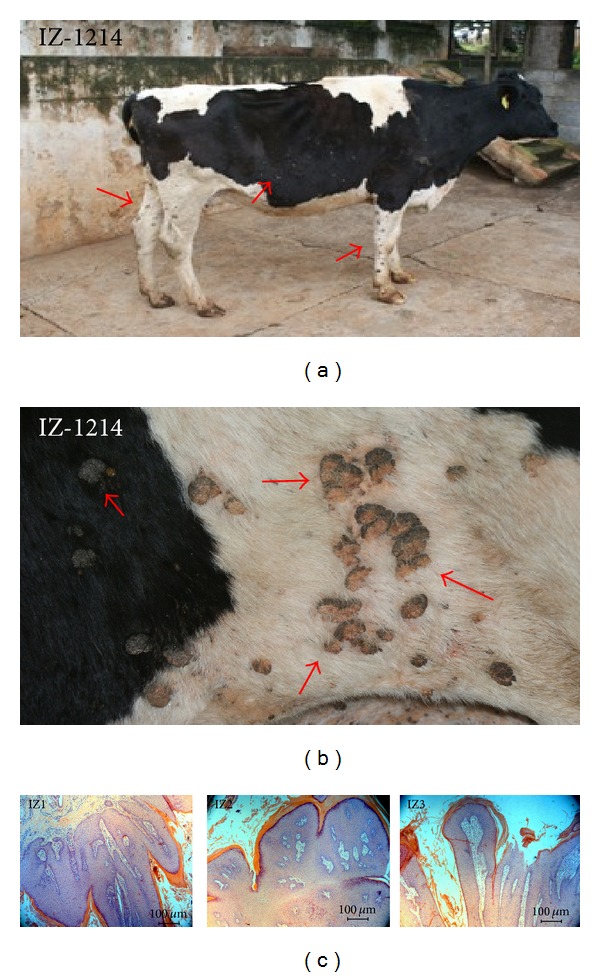
(a) Analyzed animal IZ-1214 with disseminated cutaneous papillomatosis. (b) Gross aspect of the papilloma lesions with a cauliflower-like appearance. (c) Histological preparation (hematoxylin and eosin staining, or HE) of the collected lesions (IZ1, IZ2, and IZ3) indicating characteristic hyperkeratosis, acanthosis, and papillomatosis with dermis proliferation in HE preparation (100x).

**Figure 2:**
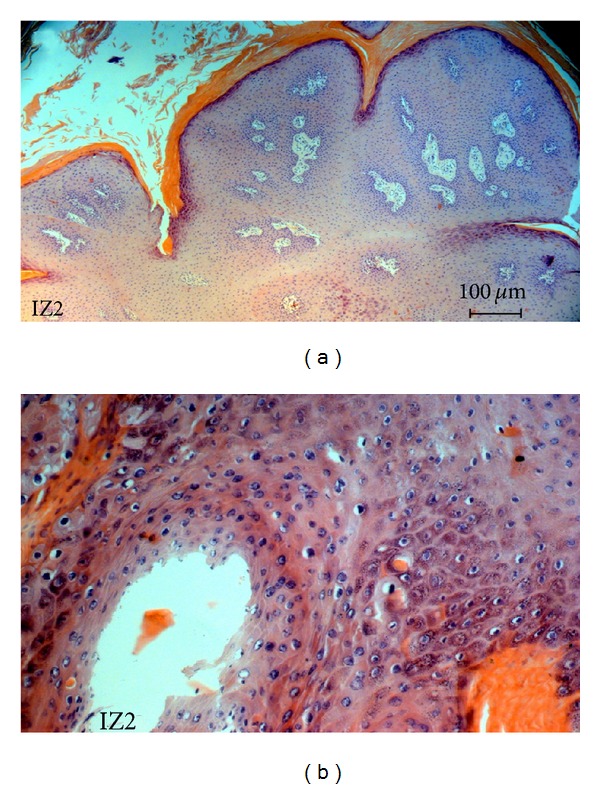
(a) Histopathology of a wart biopsy: detailed aspect of the IZ2 lesion exhibiting characteristic hyperkeratosis, acanthosis and dermal proliferation, indicated by arrows (100x). (b) Presence of koilocytosis.

**Figure 3:**
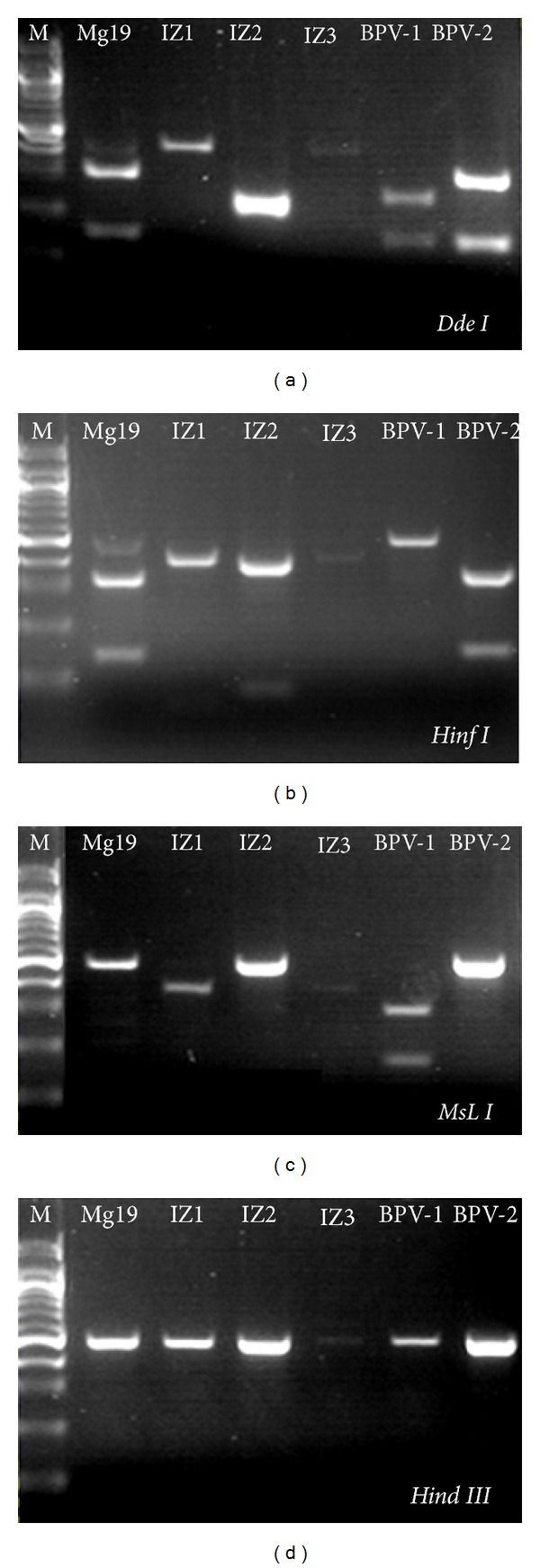
RFLP performed for restriction enzymes *Dde*I*, Hinf*I*, MsL*I, and* Hind*III. As BPV-1 and BPV-2 are the commonly associated with fibropapillomas, their L1 FAP amplicons were generated and digested as positive controls. IZ1, IZ2, IZ3 amplicons are from three different lesions of the same animal (IZ-1214). Mg-19 sample is from another bovine, clinically affected with cutaneous papillomatosis. Molecular marker: 100 bp ladder (New England Biolabs, Ipswich, UK).

**Figure 4:**
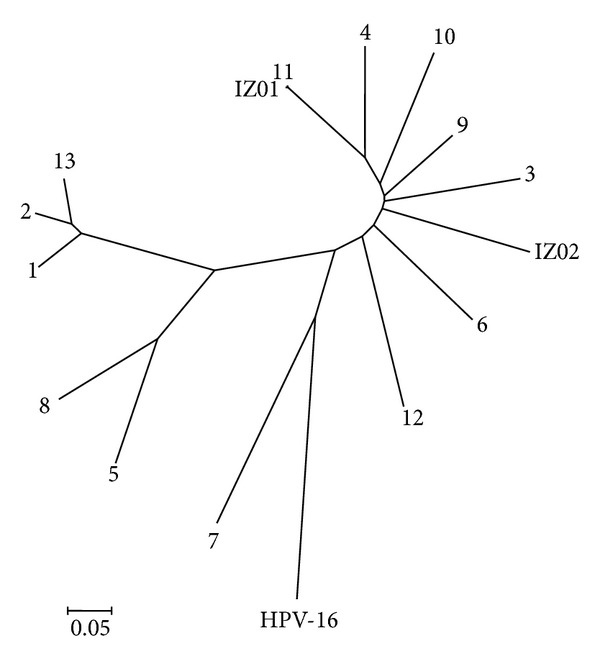
Neighbor-joining phylogenic tree constructed with 2000 bootstrap replications, using partial L1 nucleotide sequences, indicating the putative type BAPV-3 in IZ2 sample, originally described in Japan (accession: AY300819). The analysis of the other two samples, IZ1 and IZ3, demonstrated the presence of an identical sequence in both lesions, sharing 99% percent of similarity with the recently described BPV-11 type (accession: AB543507.1). As IZ3 sequence is identical to IZ1, it will not be shown here.

*In silico *analyses of the restrictionsites in FAP L1 fragments of 20 deposited BPVs sequences (from BPV-1 to BPV-13) revealed no intratype variation associated with the relative cut positions for the four enzymes employed (Table 1). Macroscopic and histological evaluations identified the collected lesions as cutaneous fibrous papillomas (Figures 1 and 2). From all the three lesions studied, two- (IZ1 and IZ3) RFLP profiling suggests the presence of BPV-11. On the other hand, IZ2 sample gene rated a profile which could not match with any of the thirteen characterized viral types (Figure 3, Table 1). In accordance with these results, DNA sequencing and BLAST analysis of IZ2 indicated a rare putative type (BAPV-3), originally described in Japan [16]. Furthermore, the DNA sequencing of other two samples confirms them as the recently described BPV-11 [10]. These sequences were deposited in GenBank (access numbers: HQ435675 and HQ612180). The phylogenetic reconstruction using this partial genomic sequence allowed its comparison with other sequences of *Papillomaviridae *family members (Figure 4). 

## 4. Discussion

Originally, the recently characterized BPV-11 was described with the employment of a *Xipapillomavirus* consensus primer [10]. Here, the same type using the FAP generic primer could be detected, indicating this set as an effective alternative for the identification of BPVs. Other than that, we described the simultaneous presence of two BPV types in three different wart samples, obtained from a chronically affected animal with disseminated papillomatosis. 

The typed BPV-11 and BAPV-3 samples have a fibrous aspect, with a similar fibropapilloma histology that is not commonly associated with the exclusive infection of the keratinocytes, commonly attributed to the Xi BPVs. In a previous report [10], Hatama and others discussed the “uncertain nature of BPV-11 tumorigenicity” since BPV-11 was first diagnosed in a fibropapilloma lesion in which the BPV-1 was also detected. Despite the eventual limitations of the diagnostic methods employed, we could detect the same viral type in at least two different cauliflower-like lesions, with a suggestive fibrous core. In addition to Hatama's report, our data also link the BPV-11 with cutaneous papillomas, indicating it as a pathogenic type. Furthermore, the putative type BAPV-3, described by Ogawa and others [16], was detected just once from a skin papilloma sample, without histological description, being genetically associated with the BPV-3, -4, and -6, or Xi BPVs. 

The L1 gene has taxonomical relevance due to its high degree of conservation, which can be accessed with the use of generic primer sets. In accordance with this, the restriction sites located in FAP fragments appears to be maintained, without intratype variations for the restriction enzymes employed, indicating that phylogenetic studies comparing BPVs and other members of the *Papillomaviridae* family are possible using a relatively short DNA sequence.

Since the early nineties, methods based on PCR-RFLP analysis within the L1 gene of the human papillomavirus (HPV) have been used for viral typing and infection diagnosis from a variety of sources, including cervical samples, fresh and paraffin-embedded tissues [34, 35]. Particularly, the PCR-RFLP method is useful to identify coinfections due to its sensitivity and specificity [36].

## 5. Conclusion

As an easy, rapid, and cost-effective assay, the PCR-RFLP represents a less-laborious approach than DNA cloning and sequencing, being an alternative as a first-line screening test, both for the diagnosis of an already classified virus type as to indicating the needing of DNA sequencing due to mixed and/or unknown digestion profiles. In a previous survey in Paraná State, Brazil, Claus and others [26] suggest that the occurrence of multiple or mixed BPVs infection may be widespread throughout Brazilian cattle herds and may occur in other Brazilian geographical regions. In agreement, our findings support these results and reinforce the notion that multiple papillomaviruses infections, with a significant pathogenic potential, can be as frequent in cattle as in human hosts [26].

To the best of our knowledge, the restriction map employed here is the first to be produced specifically for the screening and typing of BPVs. Our findings also point to the ubiquity of BPVs dissemination since two relatively rare, new described BPV types, which were first characterized in Japan, were also detected in Brazil.

## Figures and Tables

**Table 1:** L1 FAP segment digestion profiles of the BPVs-1 to -13 for *Dde*  I*, Hinf *I*, MsL *I  and *Hind*III.

	L1 FAP Fragment	*Dde*I	*Hinf*I	*MsL*I	*Hind*III
BPV-1	475 bp	264	329	301	475
		159	146	174	
		52			

BPV-2	475 bp	316	329	475	475
		159	146		

BPV-3	473 bp	319	473	373	473
		154		87	
				13	

BPV-4	469 bp	324	469	469	230
		145			182
					87

BPV-5	469 bp	469	254	469	469
			145		
			70		

BPV-6	472 bp	321	384	373	472
		151	88	99	

BPV-7	484 bp	403	271	484	484
		81	119		
			94		

BPV-8	469 bp	469	317	469	322
			152		147

BPV-9	469 bp	316	344	469	469
		153	125		

BPV-10	472 bp	319	381	373	472
		90	91	99	
		63			

BPV-11	475 bp	420	407	370	475
		55	68	105	

BPV-12	469 bp	351	317	469	469
		118	152		

BPV-13	475 bp	316	329	475	475
		109	146		
		50			

Reference Sequences:

BPV-1_NC_001522.1, BPV-1_X02346.1, BPV-2_M20219.1, BPV-2_X01768.1,

BPV-3_AF486184.1, BPV-3_AJ620207.1, BPV-3_NC_004197.1, BPV-4_X05817.1,

BPV-5_AF457465.1, BPV-5_NC_004195.1, BPV-6_AJ620208.1, BPV-7_DQ217793.1,

BPV-7_NC_007612.1, BPV-8_EB_DQ098917.1, BPV-8_NC_009752.1, BPV-9_AB331650.1,

BPV-10_AB331651.1, BPV-11_AB543507, BPV-12_JF834523, BPV-13_JQ798171.
